# Longitudinal Muscle Biopsies Reveal Inter- and Intra-Subject Variability in Cancer Cachexia: Paving the Way for Biopsy-Guided Tailored Treatment

**DOI:** 10.3390/cancers16051075

**Published:** 2024-03-06

**Authors:** Panagiotis Filis, Nikolaos P. Tzavellas, Dimitrios Stagikas, Christianna Zachariou, Panagiotis Lekkas, Dimitrios Kosmas, Evangelia Dounousi, Ioannis Sarmas, Evangelia Ntzani, Davide Mauri, Anastasios Korompilias, Yannis V. Simos, Konstantinos I. Tsamis, Dimitrios Peschos

**Affiliations:** 1 Department of Medical Oncology, School of Medicine, University of Ioannina, 45110 Ioannina, Greece; 2 Department of Hygiene and Epidemiology, School of Medicine, University of Ioannina, 45110 Ioannina, Greece; entzani@uoi.gr; 3 Department of Physiology, School of Medicine, University of Ioannina, 45110 Ioannina, Greecedimitriosstag@gmail.com (D.S.); chzachar@uoi.gr (C.Z.); ktsamis@uoi.gr (K.I.T.);; 4 Department of Orthopaedic Surgery, School of Medicine, University of Ioannina, 45110 Ioannina, Greece; 5 Department of Nephrology, School of Medicine, University of Ioannina, 45110 Ioannina, Greece; evangeldou@gmail.com; 6 Department of Neurology, School of Medicine, University of Ioannina, 45110 Ioannina, Greece; 7 Center for Evidence-Based Medicine, Department of Health Services, Policy and Practice, School of Public Health, Brown University, Providence, RI 02912, USA

**Keywords:** cancer cachexia, longitudinal, muscle biopsies, biomarker, pre-clinical study

## Abstract

**Simple Summary:**

Currently, muscle testing remains underutilized in cancer cachexia research, since muscle biopsies are mainly performed at the end of experimental protocols. We aimed to introduce the concept of longitudinal muscle testing for cancer cachexia by initially demonstrating its feasibility and safety for the test subjects. Following that, we tested the hypothesis on a tumor-bearing rat model. Results were indicative of heterogeneity in cancer cachexia manifestation between different subjects and throughout the disease course. There is an abundance of researched pathways and mechanisms of cachexia, as well as interventions to target them. Thus, moving forward, developing biomarkers to suggest “what to target and when to do it” is essential. Sequential muscle biopsies can serve as a promising tool to guide personalized precision treatment for cancer cachexia, as well as to monitor the disease evolution and response to therapy.

**Abstract:**

In the rapidly evolving landscape of cancer cachexia research, the development and refinement of diagnostic and predictive biomarkers constitute an ongoing challenge. This study aims to introduce longitudinal muscle biopsies as a potential framework for disease monitoring and treatment. The initial feasibility and safety assessment was performed for healthy mice and rats that received two consecutive muscle biopsies. The assessment was performed by utilizing three different tools. Subsequently, the protocol was also applied in leiomyosarcoma tumor-bearing rats. Longitudinal muscle biopsies proved to be a safe and feasible technique, especially in rat models. The application of this protocol to tumor-bearing rats further affirmed its tolerability and feasibility, while microscopic evaluation of the biopsies demonstrated varying levels of muscle atrophy with or without leukocyte infiltration. In this tumor model, sequential muscle biopsies confirmed the variability of the cancer cachexia evolution among subjects and at different time-points. Despite the abundance of promising cancer cachexia data during the past decade, the full potential of muscle biopsies is not being leveraged. Sequential muscle biopsies throughout the disease course represent a feasible and safe tool that can be utilized to guide precision treatment and monitor the response in cancer cachexia research.

## 1. Introduction

Cachexia, originating from the Greek words ‘kakos’ and ‘hexis’, implies a state of poor function and appearance. Indeed, cachexia has been established as a debilitating multifactorial hypercatabolic syndrome, which is primarily characterized by progressive skeletal muscle mass depletion [1]. This condition has been strongly associated with cancer, noted to occur in up to 80% of the cases in advanced disease, and to be responsible for detrimental effects on quality of life [2,3] and patient mortality [4,5]. There is, thus, a clear need to develop and refine a framework to effectively identify and manage the continuum of cancer cachexia, which consists of pre-cachexia, cachexia, and refractory cachexia [1].

The established knowledge of the multitude of alternating mutations and pathways that define cancer, as well as the requirement to identify and target these specific changes throughout the disease course have shifted the attention of the contemporary cancer care towards longitudinal biopsy-driven personalized treatment and patient monitoring [6]. Similarly, skeletal muscle wasting in cancer cachexia is governed by a plethora of molecular alterations encompassing inflammation, protein metabolism, apoptosis, and decreased tissue regeneration [5], as well as several recognized pathways such as ubiquitin-mediated proteasome degradation, autophagy, and calcium-activated protease calpains, which constitute the main routes leading to protein degradation [7]. However, biomarker research in the field is mostly oriented around the plasma and tumor samples [8,9,10], while muscle biopsies are yet to be fully utilized as a potentially valid biomarker, since even in the pre-clinical experimental setting the muscle samples are mainly collected only following euthanasia [11,12].

**Figure 1:**
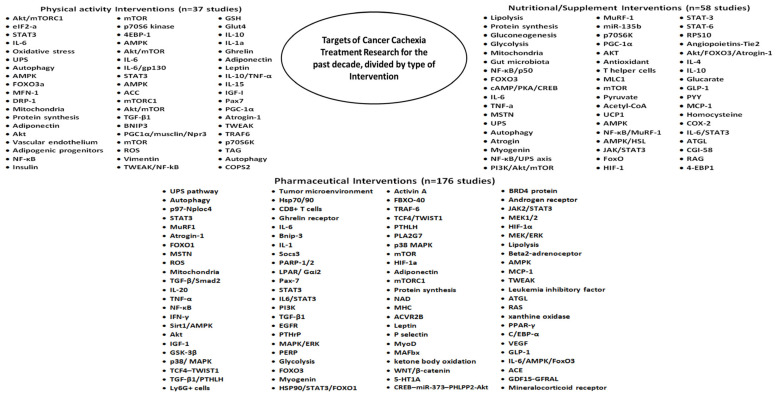
Targets of cancer cachexia treatment research divided by type of intervention. Results derived from 271 pre-clinical studies that were published during the past decade. There is an abundance of researched pathways and mechanisms of cachexia, as well as interventions to target them. Moving forward, the real challenge lies in developing biomarkers to suggest “what to target and when to do it”.

During the past decade, pre-clinical cancer cachexia research uncovered a multitude of robust targets, as well as proposed corresponding pharmaceutical, nutritional, and exercise interventions to successfully ameliorate the disease (Figure 1). The ongoing challenge is the development of valid biomarkers for cancer cachexia to prognosticate, diagnose, and monitor the disease, as well as to optimally guide treatment [13]. Cancer cachexia may demonstrate a heterogeneous manifestation and evolution patterns among individuals. We hypothesize that longitudinal muscle biopsies could reliably capture this variability, as well as guide individualized treatment based on the findings of the muscle tissue analysis at different time-points. Therefore, in this study we aimed to assess the feasibility and safety of consecutive muscle biopsies in rodents, in order to facilitate the introduction of longitudinal muscle testing in cancer cachexia research protocols as a possible prognostic, predictive, and monitoring biomarker, as well as a guide for tailored treatment. Additionally, we applied this technique for monitoring of cachexia in a leiomyosarcoma rodent tumor model.

## 2. Materials and Methods

The experimental procedures were approved by the Animal Research Committee of University of Ioannina Medical School (permit number: EL33-BIOexp01-14567) and conducted in accordance with the ARRIVE guidelines for animal experiments. All experimental subjects were male to prevent potential gender-related heterogeneity in results.

### 2.1. Feasibility and Safety Assessment of Sequential Muscle Biopsies

In order to assess the feasibility and safety of longitudinal muscle biopsies, as well as exclude the possibility for the induction of cachectic-like phenotypes by this procedure, we applied the protocol to 5 healthy rats (Sprague Dawley, 5–6 months old) and 5 healthy mice (CD1, 12–14 weeks old). The subjects were observed for a total of 15 days. All subjects were housed individually at standard housing temperatures (22 ± 1 °C). All subjects were maintained on a standard light–dark cycle (6 a.m.–6 p.m.) and were allowed ad libitum access to water and food. New food pellets were given each day in a ceramic bowl, while food from the previous day was measured and discarded. On the 5th day the first muscle biopsy was performed on the right leg and on the 10th day the second biopsy was performed on the left leg. Anesthesia was performed with intraperitoneal injection of 10 mg/kg xylazine and 100 mg/kg ketamine. The incision was approximately 1.5 cm in length. The biopsies were performed by an orthopedic surgeon with expertise in animal models and muscle biopsies (DK). The duration of the evaluation, as well as the designation of the biopsy time-points, were based on the duration of contemporary high-impact pre-clinical cancer cachexia protocols and the time-points at which preventive and interventional treatment is initialized, respectively [14]. Evaluation of the rodents was performed daily by one experienced investigator (NT) at a standard time, and every second day by an independent investigator (DP) who was blinded to the first investigator’s assessments.

Several assessment questionnaires from the published literature were utilized in order to ensure the reliable interpretation of outcomes. The daily evaluation forms were constructed based on four compartments:

- Exclude the induction of cachexia-like characteristics: weight loss observed in daily body weight measurements; daily observation of the subjects’ gait pattern (this parameter is of interest since it could lead to possible immobilization and muscle atrophy); observation of eating and drinking patterns (in order to perform this assessment we observed feces and urine [15]).
- Body condition and behavioral scoring as described in detail by Paster et al. [16] (11–13: healthy, 6–10: morbidity, ≤5: consider euthanasia), including the following: appearance, natural behavior, provoked behavior, body condition score.
- Pain score index/ethogram [17,18] including the following: cat pain index score, eyes pain index score, coordination/posture pain index score, overall condition pain index score.
- Grimace Scale for rats [19] and mice [20] for assessing pain.

### 2.2. Cell Culture and Generation of Tumor Model

In this study, we utilized leiomyosarcoma cells that were isolated from tumors histologically identified as leiomyosarcoma in Wistar rats and stored in liquid nitrogen until use. All experiments were performed with mycoplasma-free cells. Cells were maintained in Dulbecco’s Modified Eagle Medium low glucose supplemented with 10% (*v*/*v*) heat-inactivated fetal bovine serum, 1% glutamine, and 1% penicillin/streptomycin with incubators maintained at 37 °C and 5% CO_2_. Cells were sub-cultured in preparation for subcutaneous implantation to animals until the desired concentration of 5 × 10^6^ cells.

Subcutaneous implantation with injection of 5 × 10^6^ cells was performed in five rat subjects. Cells were suspended in phosphate-buffered saline of 200 μL final volume and injected without anesthesia. Subcutaneous injection was performed dorsally to the region of spine. Muscle biopsies were obtained from the legs, as described above in the healthy rats.

### 2.3. Tissue Collection and Histology

Our only means of tumor detection was by palpation. In pilot experiments we have noted that, for this model, the tumor starts being palpable approximately by the 40th day after cancer cell injection (Day 0). Therefore, we considered the 30th day as the appropriate time-point for the first muscle biopsy (early stages of cancer development) and the 40th day as the time-point for the second muscle biopsy (established cancer development). Following that, the tumor undergoes rapid visible growth leading to the demand for euthanasia by the 47th day. Euthanasia was performed by CO_2_ inhalation and a final (third) muscle sample was collected. The muscle biopsies were performed as described in the feasibility assessment protocol and the samples which were collected from the healthy rats were used as controls.

### 2.4. Muscle Biopsy Processing

After the surgery, the muscle tissue was placed on a tissue holder and carefully aligned to ensure transverse sectioning on the microtome. Subsequently, it was incubated for 15 s in 3,5-Dimethyl-Butane that had been pre-frozen in liquid nitrogen. Following this, the tissue was promptly placed in deep freezing at −80 °C, where the muscle biopsies were kept until the sectioning process. Sections with a thickness of 8 μm were cut using a freezing microtome at a temperature of −20 °C. These sections were then subjected to standard histological staining, including hematoxylin and eosin staining, as well as modified Gomori trichrome stain. Additionally, immunohistochemistry was performed using antibodies targeting myosin heavy chain fast, slow, neonatal, and major histocompatibility complex. The assessment of the biopsies was conducted using a light microscope by two experienced and independent researchers.

## 3. Results

Five healthy rats and five healthy mice were followed-up for 15 days. In the 5th and 10th day, muscle biopsies were performed on the right and the left leg, respectively. Results are summarized in Table 1.

None of the rats exhibited weight loss (Kruskal–Wallis test, *p*-value = 0.998) or alteration of eating habits throughout the course of the protocol. They kept a healthy appearance and interactive behavior, as well as a strong demeanor, which were not affected by the two surgeries. Careful evaluation of facial actions based on the rat grimace scale showed moderately present disturbance in one subject, only at the day following the second biopsy. A slight limp was noted in two subjects following the first biopsy and in three subjects following the second biopsy. This limp, which was evident only at the 6th and 11th day (only at the days following the biopsy), occurred on the side of the biopsy and was not accompanied by lowering of the reflexes or mobility of the rat.

Regarding the mice, again no weight loss (Kruskal–Wallis test, *p*-value = 0.783) or alteration of eating habits occurred. Only one mouse presented weight loss above 10%. The subjects kept a healthy appearance and interactive behavior with high mobility at every time-point throughout the course of the protocol. All the subjects were assessed to have surgery-related limping and moderately present disturbance according to the mouse grimace scale on the 6th and 11th day of the study. These findings were not detected at the 7th and 12th day.

To determine the feasibility of the protocol in tumor-bearing models, five rats were injected with cancer cells (Day 0). Muscle biopsies were performed on the 30th and 40th day. All the subjects had palpable tumors by the 43th day. Euthanasia was performed on the 47th day and a final muscle biopsy was retrieved. In complete concordance with the feasibility assessment results, only two tumor-bearing rats presented with a slight limp immediately following surgery that lasted for a day, without any further surgery-related functional restrictions. All the rats progressively exhibited a frailer demeanor, as well as muscle wasting by clinical examination with palpation. The subjects presented with a progressive reduction in food intake, a typical characteristic of cachexia, which was especially prominent at the late stages of the experiment. The tumor-bearing rats did not exhibit significant weight changes despite the reduction in muscle loss, due to the rapid growth of tumor size that was observed in all cases.

**Figure 2:**
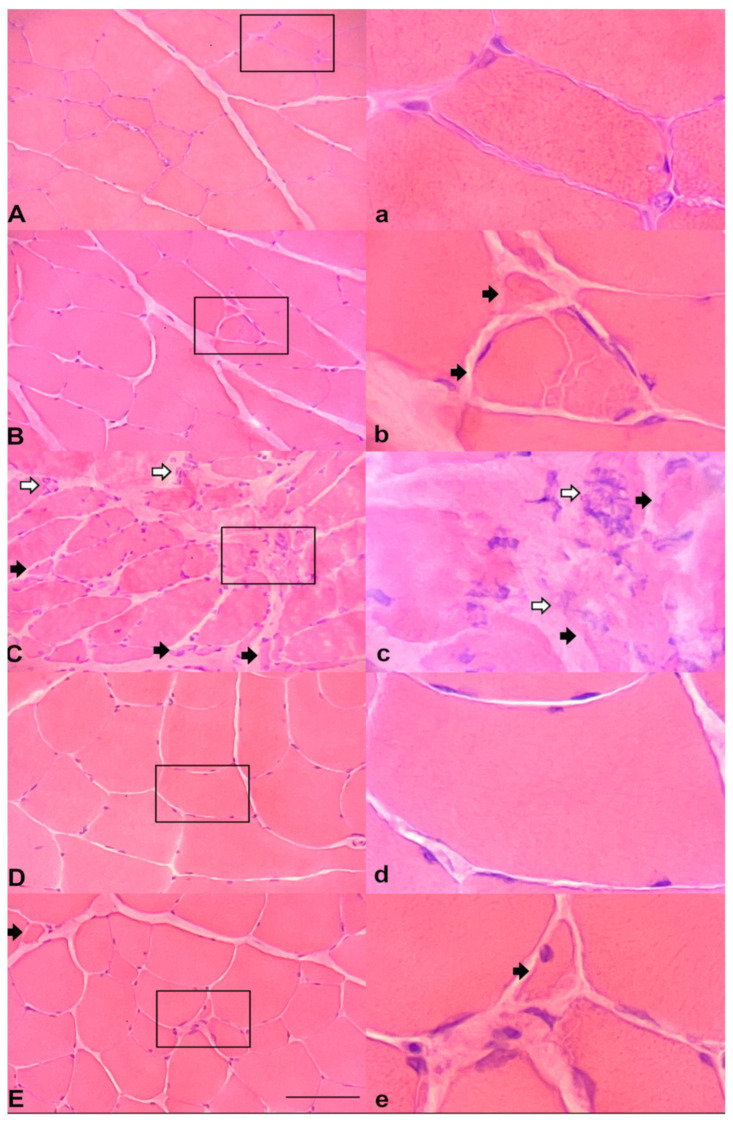
Muscle biopsy sections stained with hematoxylin and eosin. The left-side figures (**A**–**E**) depict muscle biopsy sections stained with hematoxylin and eosin. Corresponding areas of interest (marked within a black frame) are magnified in the right-side photomicrographs ((**a**–**e**), respectively). Atrophic muscle fibers are marked with black arrows and leucocyte infiltration with white arrows. (**A**) Control case (injected with saline): no evidence of muscle atrophy or leucocytic infiltration. (**B**) Case with mild muscle fiber atrophy (30 days after tumor cell injection). (**C**) Case with significant muscle fiber atrophy and leucocytic infiltration (30 days after tumor cell injection). (**D**) Case without muscle atrophy or leucocytic infiltration (30 days after tumor cell injection). (**E**) The same case as in (**D**) with mild muscle atrophy (40 days after tumor cell injection). Scale bar is 50 μm.

Microscopic evaluation of the muscle biopsies showed heterogeneity among findings in different subjects and at different time-points. Muscle atrophy, which is a typical characteristic of cachexia, was present in subjects at varying intensities and time of onset. Figure 2 presents the most notable findings. A subject injected with saline served as a control to facilitate comparisons (Figure 2a). One case presented mild muscle fiber atrophy at the time of the first biopsy (Figure 2b), without any evidence of frailty by clinical examination, and the atrophy became even more evident in the sequential biopsies. This case showcases how muscle biopsy findings can precede clinical manifestation of the disease. A different subject, again at the point of the first biopsy, presented significant muscle fiber atrophy accompanied by leucocytic infiltration (Figure 2c), underscoring the differences when compared to Figure 2b and supporting the presence of inter-subject heterogeneity of the muscle involvement at the same time-points. Finally, another subject, at the time of the first biopsy, did not present noteworthy findings (Figure 2d), while in its second sequential biopsy mild atrophy was noticed (Figure 2e), supporting the presence of chronological progression of the muscle disease and highlighting the requirement for continuous monitoring.

## 4. Discussion

**Figure 3:**
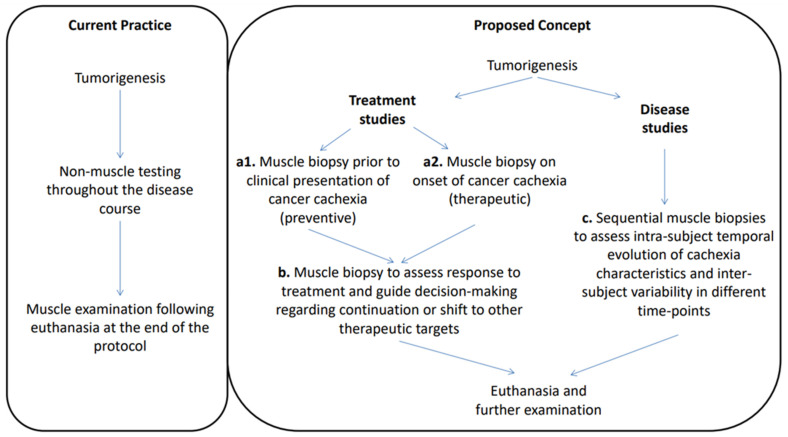
This figure demonstrates the collective potential of the proposed concept in contrast to the current cachexia research practice. We have demonstrated that the muscle involvement varies between subjects and at different time-points (Figure 2). Longitudinal muscle biopsies throughout the disease course could serve as a promising and reliable biomarker to monitor the disease and guide personalized therapy based on the biopsy findings. For protocols that study treatment interventions: (**a1**) early muscle biopsies can detect cachexia in its early transcriptional/molecular and cellular level, before its clinical manifestation, facilitating initiation of targeted treatment to prevent disease progression; (**a2**) in established cancer cachexia, muscle biopsy can guide individualized target-specific treatment, alleviating the disadvantage of “blindly” targeting possibly responsible pathways; (**b**) muscle biopsies following therapy initiation can evaluate the response to the specific treatment and guide decision making towards either continuation or shift to another target. For studies that aim to map the cachexia characteristics in a variety of tumors and settings, sequential muscle biopsies can do the following: (**c**) provide insights into the molecular and cellular changes occurring in the muscle tissue during the disease; assess the evolution of cancer cachexia in each individual subject (intra-subject temporal evolution), as well as potentially highlight differences between subjects (inter-subject variability); identify potential biomarkers associated with the onset and progression of cancer cachexia.

In this study, we introduced the concept of longitudinal muscle biopsies (Figure 3), assessed its feasibility, and presented promising initial results that support it. Despite the plethora of targets and interventions in cachexia research, their reflection in the clinical setting of cachexia treatment has been negligible [21]. There is no paucity of means, but the real challenge lies on what to target and when it should be done. Our initial results demonstrate heterogeneity of cancer cachexia manifestation throughout the course of the disease. The benefit of sequential testing is the evaluation of intra- and inter-subject variability at different time-points of the disease. This knowledge can potentially facilitate an in-depth understanding of the heterogeneous underlying mechanisms of cachexia during different stages of development, as well as guide individual subject-informed and specific decision making. Thus, we propose longitudinal biopsies as a tool to study cancer cachexia, as well as to guide and monitor treatment, facilitating its delivery in an individualized and timely manner.

Cachexia treatment research in pre-clinical models has grown exponentially during the past decade, consisting of pharmaceutical, nutritional, and exercise interventions (Figure 1). Even though the muscle is the primary tissue of interest for these therapeutic interventions, it is being tested only once following termination of an experiment. However, this established framework may contain limitations, since the dynamic evolution of the tumor could also imply continuous transformation of the character of cachexia. In the early 2000s, two pathways for cachexia were underscored, characterized by a decrease in transcription of myosin heavy chain originating from TNF-a or by an increase in ubiquitination of myosin attributed to IL-6 [22]. This knowledge could have translated into three separate conclusions at the time: either only one pathway exists in each cachexia experimental model; both pathways can exist with one being more dominant in each different model; or even more interestingly both pathways can exist, but their upregulation is interchangeable throughout the disease course.

To further add to this thought process, treatment interventions have been shown to generate varying results depending on the time-point of administration [14,23]. While this fact is in line with the general notion regarding disease prevention to combat it at its early stages [24], it also suggests that the loss of treatment efficacy could be attributed to differentiation of regulatory processes throughout the course of cancer cachexia. Currently, in the clinical guidelines for cancer cachexia, no pharmaceutical interventions can be recommended since results from trials are scarce and indecisive [21]. Through this work, we propose that leveraging longitudinal muscle biopsies in experimental models, as well as possible future introduction in the clinic, could have a dual benefit for cancer cachexia therapy protocols, contributing to further evolution of this research field. Firstly, the initial muscle biopsy can drive the treatment decision for cancer cachexia, which will be tailored to the individual results that are retrieved. Secondly, sequential biopsies allow for the continuous monitoring of response to the treatment at different time-points of the disease course, and give the advantage of a guided therapy shift in case of differentiation of the disease character.

The development of predictive and monitoring biomarkers for cancer cachexia has risen as a requirement of paramount importance [25]. Potential biomarkers exist in the serum, the muscle tissue, the adipose tissue, and the genome [26]. Several metabolomics [27] and proteomics profiling [28], as well as micro-RNA studies [29,30] and transcriptome analyses [31], are emerging to identify circulating and muscular markers of wasting process during cancer cachexia. Notably, a recent study applied a metabolomics approach in the serum and muscle following sacrifice in different time-points in a murine model of cachexia [32]. Although these muscle biopsies were singular, it could be implied that this study indirectly demonstrated the plethora of information that can originate from the coupling of both sequential muscle biopsies and serum sample analyses. Singular sampling could be indicative of inter-subject variability, but is unable to assess intra-subject heterogeneity and profiling differentiation. Interestingly, the benefits of longitudinal transcriptomic analysis for monitoring of age-related muscle pathology have been recently described [33]. The research of sarcopenia, a clinical entity that presents similarities to cachexia and is characterized by reduced muscle strength and low muscle mass [34], could also be complemented by sequential biopsies in order to monitor the ongoing changes of muscle tissue.

The role of noninvasive monitoring using imaging modalities, such as computed tomography, magnetic resonance imaging, (18F) fluoro-2-deoxy-D-glucose (18FDG) positron emission tomography, and dual-energy X-ray absorptiometry, should not be neglected [35]. Non-invasive approaches hold a clear benefit regarding applicability and reproducibility. Expanding the role of imaging phenotyping for the temporal characterization of cachexia, the assessment of high-risk individuals, and the monitoring of treatment is steadily gaining ground as a reliable approach. In fact, longitudinal imaging-based monitoring of cancer cachexia development, both in mouse models and immune checkpoint inhibitor-treated patients, has recently demonstrated favorable results [36]. Moreover, imaging studies can offer valuable information to calculate energy expenditure in patients with cancer, which consequently leads to negative energy balance and can be an essential determinant for cachexia [37]. As we are already traversing the artificial intelligence era [38], the potential linking of longitudinal imaging phenotyping and sequential muscle biopsies testing, as well as serum biomarker analyses, holds great promise for the future perspectives of cancer cachexia research.

A limitation of our study was that despite successfully obtaining muscle biopsies from the tumor-bearing rats, we were restricted only to results for immunohistochemistry without transcriptome analyses and other “omics” approaches which would offer essential information. Differential expression of cachexia-related signaling pathways, such as FoxO1/MuRF1/Atrogin-1, at different time-points throughout the disease course, would be of special interest. However, the main aim of this study was to introduce the concept of longitudinal muscle biopsies for cancer cachexia and to assess the feasibility of this framework in healthy and tumor-bearing pre-clinical subjects, as well as to demonstrate initial results of heterogeneity in cachexia manifestation and evolution, so that its evidence-based implementation and evaluation would be facilitated from future experimental studies. Another limitation was that the body mass loss of the tumor-bearing rats at the final stages of the experiment could not be reliably measured because of the large size of the tumor, despite the muscle wasting that was evident by clinical examination and microscope assessment, as well as the reduced food intake. Application of this framework to a variety of tumor models by future studies is essential as we move forward.

## 5. Conclusions

In conclusion, muscle biopsies have the potential to become an important companion tool to the constantly evolving clinicopathological, imaging, and biomarker landscape of cancer cachexia. Thus, it is highly suggested that the concept of longitudinal muscle biopsies to identify the disease, monitor the progression and response to treatment, as well as guide tailored cachexia therapy, should be implemented in the scientific protocols of cachexia research centers for a variety of settings, in order to pave new avenues towards a potentially more holistic cachexia management.

## Figures and Tables

**Table 1:** Results from the feasibility and safety assessment of longitudinal muscle biopsies with three different questionnaires in healthy rat and mouse subjects.

Variable	Scoring System/Type of Measurement	Notable Assessment Comments
Time of the Day	Every measurement at 12:00 p.m.	
Body Weight	Measured in grams	No weight loss was noted for rats. Only one mouse presented weight loss above 10%.
Is the gait normal	Score = 1: Yes, Score = 0.5: Slight limp, Score = 0: No.	Rats: two subjects following the first biopsy and in three subjects following the second biopsy (limp lasted only for one day) Mice: All had a slight limp only the day following the surgeries, only at the site of the biopsy each time
Signs of eating/drinking	2 points: feces and urine observed, 1 point: minimal feces and/or urine output, 0 points: no signs of feces and/or urine.	No alteration of eating habits was noted
**Body condition and behavioral scoring**		
Appearance	Normal (Score = 2): bright eyes, shiny well-groomed hair coat, erect ears, pink mucous membranes; abnormal (Score = 1): unkempt hair coat, dull fur; abnormal (Score = 0): hunching, piloerection, soiled fur, dry eyes.	Normal throughout the protocol
Natural Behavior	Normal (Score = 3): active, interactive in environment, looks up to the observer; normal (Score = 2): Slight decrease in activity, less interactive, disregards the observer; abnormal (Score = 1): pronounced decrease in activity, isolated in corner of cage; abnormal (Score = 0): possible self-mutilation, hyperactive, or immobile.	Normal throughout the protocol
Provoked Behavior	Normal (Score = 3): quickly moves away; normal (Score = 2): slow to move away or exaggerated response; abnormal (Score = 1): moves away after sort period of time; abnormal (Score = 0): does not move or reacts with excessively exaggerated response.	Normal throughout the protocol
Body Condition Score	Emaciated (Score = 1): no palpable fat over the sacroiliac region, severely reduced muscle mass, with prominent vertebrae and iliac crests; thin (Score = 2): thin with some fat deposition and muscle mass but less than that palpated in mice with a score of 3, also had visible iliac crests; normal (Score = 3): had easily palpable fat pads, reduced definition of vertebral bodies, palpable but not visible iliac crests, and thick prominent muscle mass; overweight (Score = 4): difficulty in palpating iliac crests, difficulty in assessing vertebral definition, and prominent fat pads overlying muscled areas; obese (Score = 5): fat pads that overlaid muscle and iliac crests, thereby obscuring their presence both tactilely and visually and giving the animal’s rump a rounded appearance.	Normal throughout the protocol
Total Score	Score = 11–13: healthy, Score = 10: morbidity, Score < 5: consider euthanasia.	Score 11–13 in every subject
**Pain score index/ethogram**		
Coat pain index score	Score 0: normal, well groomed, smooth sleek hair coat; Score 1: not well groomed; Score 2: rough hair coat, dirty incision; Score 3: very rough hair coat, hair loss, dirty incision.	Normal throughout the protocol
Eyes pain index score	Score 0: open/alert; Score 1: squinted eyes; Score 2: closed.	Mice: Squinted eyes in every subject, only at the day following each biopsy
Coordination/ posture pain index score	Score 0: normal; Score 1: walk awkward or slightly hunched, still runs or moves fast; Score 2: walked hunched, walking on eggshells, does not run, walks slowly; Score 3: walks slowly with effort or only by tapping on cage; Score 4: hunched, stumbles, or must be pushed to get to move; Score 5: hunched, will not walk even when pushed.	Normal throughout the protocol
Overall condition pain index score	Score 0: normal; Score 1: rough appearance but acts fairly normal; Score 2: slightly depressed, rough appearance, or slightly agitated; Score 3: very rough or very agitated.	Normal throughout the protocol
**Rat/mouse grimace scale**		
Orbital tightening	Mouse/rat grimace scale photos (Score 0–2) Closing of the eyelid (narrowing of orbital area), a wrinkle may be visible around the eye	Rats: moderately present disturbance only in one subject, only at the day following the second biopsy Mice: moderately present disturbance in every subject, only at the day following each biopsy
Nose bulge	Mouse grimace scale Photos (Score 0–2) Bulging on the bridge of the nose, vertical wrinkles on the side of the nose	Mice: moderately present disturbance in every subject, only at the day following each biopsy
Cheek bulge	Mouse grimace scale photos (Score 0–2) Bulging of the cheeks Rat grimace scale photos (Score 0–2) Flattening and elongation of the bridge of the nose, flattening of the cheeks	Rats: moderately present disturbance only in one subject, only at the day following the second biopsy Mice: moderately present disturbance in every subject, only at the day following each biopsy
Ear position	Mouse grimace scale photos (Score 0–2) Ears rotate outwards and/or backwards, away from the face Ears may fold to form a ‘pointed’ shape, space between the ears increases Rat grimace scale photos (Score 0–2) Ears curl inwards and are angled forward to form a ‘pointed’ shape, space between the ears increases	Mice: moderately present disturbance in every subject, only at the day following each biopsy
Whisker change	Mouse grimace scale photos (Score 0–2) Whiskers are either pulled back against the cheek, or pulled forward to ‘stand on end’, whiskers may clump together, whiskers lose their natural ‘downward’ curve Rat grimace scale photos (Score 0–2) Whiskers stiffen and angle along the face, whiskers may ‘clump’ together, whiskers lose their natural ‘downward’ curve	Mice: Moderately present disturbance in every subject, only at the day following each biopsy

## Data Availability

The data that supports the findings of this study are available in the article.
